# Can we distinguish the roles of demographic and temporal changes in the incidence and prevalence of musculoskeletal disorders? A systematic review

**DOI:** 10.5271/sjweh.4018

**Published:** 2022-04-29

**Authors:** Hanifa Bouziri, Alexis Descatha, Yves Roquelaure, William Dab, Kévin Jean

**Affiliations:** 1 Laboratoire MESuRS, Conservatoire national des Arts et Métiers (Cnam), Paris, France; 2 Inserm, EHESP, Irset (Institut de recherche en santé, environnement et travail) – UMR_S 1085, Univ Angers, Univ Rennes, 28 Roger Amsler, CS 74521, 49045 Anger, Cedex 01, France; 3 MRC Centre for Global Infectious Disease Analysis, Department of Infectious Disease Epidemiology, Imperial College London, United Kingdom

**Keywords:** epidemiology, chronic disease, musculoskeletal pain, occupational health, temporal trend

## Abstract

**Objectives:**

Musculoskeletal disorders (MSD) represent a major public health issue, affecting more then 40 million European workers in 2017. The overall aging of the working population is expected to increase the burden of disease, but temporal changes in exposures or diagnosis may also drive the global trends in MSD. We therefore conducted a systematic review to summarize the evidence on the role of demographic and temporal changes in the occurrence of MSD.

**Methods:**

We conducted a systematic review of articles reporting temporal trends in MSD in the general working-age population. Only articles controlling for age in the analysis were included. The risk of bias was assessed. The main indicators extracted were age-controlled time trends in MSD incidence or prevalence.

**Results:**

Among 966 articles, 16 fulfilled the inclusion criteria, representing 23 results according to the indicators extracted. No study was found with a high risk of bias. Results presenting time trends in prevalence were found in 12 studies and incidence in 11. After controlling for age, the reported temporal trends varied, mostly between non-monotonic changes (N=12/23) and increases (N=10/23). One article also highlighted an increase among women and non-monotonic changes among men (N=1/23). Several factors other than aging were suggested to explain temporal trends in MSD, mainly trends in obesity, changing occupational exposures, and cultural factors regarding pain tolerance.

**Conclusion:**

This review shows that different kind of factors in addition to aging may contribute to varying or increasing trends in MSD. This review also highlighted the scarcity of evidence regarding time trends in the burden of MSD and their underlying causes.

Musculoskeletal disorders (MSD) affected more than 40 million workers in Europe in 2017 and were the leading contributors to disability worldwide in 2019 (1, 2). These conditions refer to a group of painful disorders of muscles, tendons, and nerves. The causes of MSD are multifactorial and notably can be induced by occupational, biomechanical and psychosocial risk factors (3). Since the 1970s, there have been many changes in working conditions, catalyzed most notably by increased digitization across a range of professions, and widespread reinforcement of preventive actions has redistributed the risk factors of MSD (4–6). There has also been an increase in employment in the service sector, which contributes to changes in the patterns of exposure to hazards at work (1). The combined effects of these occupational changes on the temporal evolution of MSD are thus challenging to assess. In addition, the aging of the workforce could have implications for the increasing risk of chronic diseases like MSD (6, 7). In particular, the rising average age of workers in many high-income countries may increase the risk of MSD in the absence of preventive action. This owes in part to degenerative phenomena linked to the aging process itself, which induces a reduction in biomechanical tolerance to repetitive and/or prolonged loading, and in part to prolonged exposure to residual biomechanical stresses and psychosocial risks accumulated during increasingly long careers (3).

Exposures to leading occupational risk factors of MSD, such as biomechanical, organizational, and psychosocial factors, have evolved heterogeneously over time (8–11). Consequently, successive cohorts of workers have not been exposed to MSD risk factors with the same intensity and frequency throughout their lives. Moreover, risk factors of MSD and their changes over time have mainly been studied individually or by family (eg, biomechanical, organizational, psychosocial), but a global view of their simultaneous change over time is still lacking. Disentangling the respective roles of age and temporal evolution in exposure to risk factors on the occurrence of MSD is thus needed to understand current trends and design adapted prevention policies (3–14). Furthermore, understanding the evolution of exposures over time while accounting for age would allow for more accurate prediction of future trends in MSD and help to prevent and control their occurrence (15, 16).

The objective of this study was to collate and review the existing evidence on the respective roles of demographic and temporal changes in the occurrence of MSD. We used the systematic search and review methodology as previously described by Grant & Booth (17). This type of review consists of combining a systematic search method with a critical review analysis and is used to answer broad questions while often incorporating multiple study designs.

## Methods

### Search strategy

The study protocol was registered in PROSPERO (CRD42020221499) (18). This protocol is consistent with the Preferred Reporting Items for Systematic Review and Meta-analysis (PRISMA) guidelines (19, 21). A detailed PRISMA 2020 checklist is provided in the supplementary material (www.sjweh.fi/article/4018), table S1. Any modification of the methods stated in the original protocol was registered in PROSPERO (see the reference mentioned before).

### Literature search

We searched four different electronic bibliographic databases for studies published between 1990 and 2020: Medline, ScienceDirect, Wiley, and Web of Science. The last source searched or consulted was checked in November 2020. Details of the search strategy used for each database are provided in supplementary table S2, including the algorithms of keywords used, the number of results and the articles preselected for screening.

### Inclusion and exclusion criteria

For the article identification step, when the databases allowed, we automatically excluded results related to topics not relevant for our search, such as studies involving animals, molecular biology, immunology studies or clinical case reports.

Then, a first round of selective screening was carried out based on titles and abstracts (step 1). Only original articles were included; conference reports, literature reviews, and editorials were excluded. At this stage, only articles that reported MSD or MSD proxy outcomes while mentioning the notion of temporal trends were included.

In the full-text assessment (step 2), articles defining MSD as a group or set of diseases localized at or around the joints (wrists, elbows, shoulders, spine, or knees) were selected. The pathologies considered here concerned the muscles, tendons and tendon sheaths, nerves, bursae, joints, ligaments, at the periphery of the joints of the upper limbs, the spine, and the lower limbs. We excluded MSD defined as a joint manifestation of organic diseases (eg, psoriasis, lupus, gout, etc.) or as the joint location of systemic inflammatory origins (eg, secondary osteoarthritis). At this step, only articles reporting temporal trends in incidence and/or prevalence in MSD while controlling for age were selected. We included studies conducted among the working-age population. Studies of people under 18 and unpaid domestic workers were excluded. The prevalence or incidence of MSD over time that only address the average over a single period were also excluded.

### Screening

The Covidence Systematic Review software allowed the selection of studies, their download, and the removal of duplicates (22). Two independent authors performed both steps 1 and 2 to assess the eligibility of studies identified in the databases. A third senior researcher resolved any conflict in article screening or full-text assessment.

### Data extraction

All articles included were read for the identification and extraction of the following characteristics: geographic location, population studied, study design and recruitment criteria, start and end date of follow-up, MSD sites (superior limbs, inferior limbs & back, or not specified), criteria used for MSD definition (either based on pain or on disability), and the method used for MSD diagnosis.

### Assessing risk of bias and quality of evidence

To assess the risk of bias across included studies, we used the RoB-SPEO (23) and the Navigator guide tool, which we adapted for our study (see supplementary material 4 for methodological details) (24, 25). The biases we assessed were selection bias, potential biases linked to misclassification of MSD, biases due to incorrectly taking confounding factors into account, and bias due to potential conflict of interest. Each article has been classified according to its level of bias (low, probably low, probably high, high). We also assessed the quality of the statistical trends tested by using the following classification: satisfactory quality, probably satisfactory quality, probably unsatisfactory quality, unsatisfactory quality. Further details on the criteria and classifications used for the risk of bias and quality of evidence assessment are available in supplementary material S4.

### Analysis of the temporal trends of the occurrence of MSD

The following data were extracted from each article: the raw temporal trends of MSD prevalence and/or incidence (if reported), information concerning methods used to control for age, and MSD prevalence and/or incidence over time after controlling for age. For each article, the temporal trends in MSD prevalence and/or incidence were analyzed according to the location and severity of MSD. If an article investigated multiple types of MSD and/or addressed both temporal trends in the prevalence and incidence of MSD, we considered these results independently; therefore, the total number of results could possibly be higher than the number of studies included. We also distinguished two groups of results based on the MSD sites and criteria used for MSD definition, either based on pain or on repercussion on work and/or social life (hereafter called disability). The precise definition of MSD used in each article is provided in supplementary table S3. Temporal changes in MSD prevalence/incidence were summarized according to whether they decreased, varied non-monotonically, or increased.

### Synthesized evidence

For articles reporting both raw and age-adjusted MSD time trends, we compared findings to discern differences between them, and therefore to assess whether it would be possible to dissociate age from time in the occurrence of MSD over time. When mentioned, we summarized the interpretations and hypotheses proposed to explain observed temporal trends in MSD prevalence/incidence.

## Results

### Studies selected

**Figure 1:**
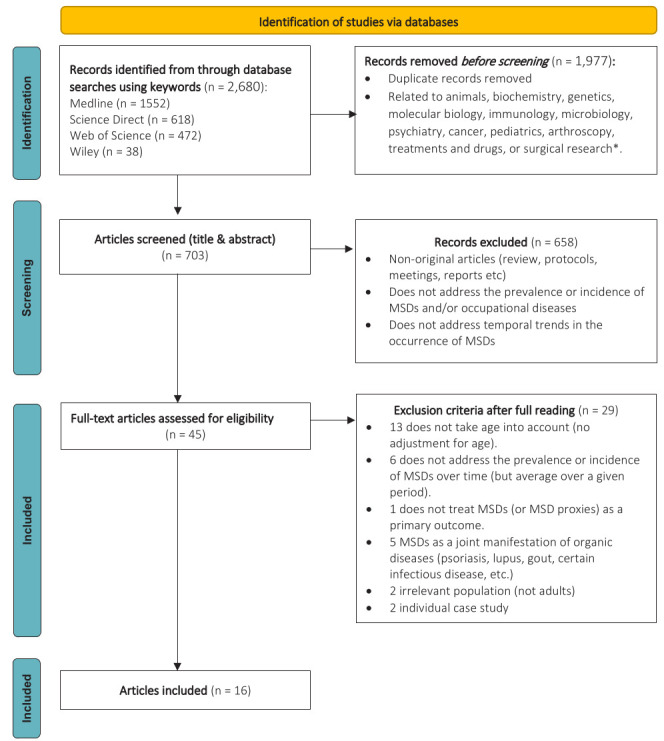
Flow chart diagram of study selection using PRISMA Flow Diagram recommendations. * Automatically excluded when the classification tools of the database allowed. Inspired by Page MJ, McKenzie JE, Bossuyt PM, Boutron I, Hoffmann TC, Mulrow CD, et al. The PRISMA 2020 statement: an updated guideline for reporting systematic reviews. BMJ 2021;372:n71. doi: 10.1136/bmj.n71. For more information, visit: http://www.prisma-statement.org

A total of 2680 study records were identified through our systematic search, of which 1977 were excluded, 335 were duplicates, and 1642 were deemed irrelevant as per automatic categorization tools provided by some databases (figure 1). A further 658 records were excluded after title and abstract screening because they did not present original results and/or did not report results based on MSD and/or did not address temporal trends in their occurrence. Of the 45 fulltext articles assessed for eligibility, 29 were classified as ineligible, 13 of which because they did not control for age in reported indicators. A total of 16 studies fulfilled all eligibility criteria and were thus included in the review.

### General study characteristics

The 16 articles included in the present review were published between 2003 and 2020 (table 1), 12 of which were published after 2010. Overall, the studies were conducted in three geographic areas: 12 in Europe (among which 5 were in Scandinavian countries), 3 in the USA, and 1 in Australia. The duration of the study period ranged from 10–55 years across studies. In 8 studies, sampled populations were used (3 cohort designs, 5 repeated cross-sectional studies), representing a total of 1 387 930 individual working-age adults. Among these, 2 focused on the male population only. The other 8 studies relied on a time-series design based on surveillance data collected within five countries and one subnational administrative area.

**Table 1:** Summary of articles reporting temporal trends in occurrence of musculoskeletal disorders (MSD).

Reference & country	Recruitment	Period	Population	MSD type	Diagnosis	Raw time trends	Time trends ^a^	Dealing with age	Interpretation for age specific time trends
**3 cohort studies**									

Guido et al, 2020 (29)Europe 17 studies	Variable	1991–2015 (24 years)	General population 660 028 individuals	All pain including MSD	Self-reported	Untreated	Prevalence: increase	Age-period-cohort (ACP)	Evolving perception of pain that can be explained by cultural or biological changes (in patients and practitioners).
Söderberg et al, 2020 (34) Sweden	National occupational health service	1977–2010 (33 years)	389 132 individuals Construction workers (male 20–60 years)	Disability pension caused by MSD	Medical exam	Incidence: variable	Incidence: variable	Stratified ages	Changes in welfare legislation (pension eligibility criteria) rather than underlying exposures.
Solomon et al, 2007 (48) UK	Household	1949–2004 (55 years)	34 486 men from rural areas	MSD-related job loss	Questionnaire	Untreated	Incidence: increased	Adjusted	Acceptance of evolving occupational diseases that can be explained by cultural changes (in patients and practitioners).

**5 repeat cross-sectional studies**									

Dick et al, 2020 (32) US	Household	2002–2014 (12 years)	General population 5135 individuals	Back pain	Questionnaire	Prevalence: Decrease	Prevalence: variable. Decrease until 54years, 55 – 64: variable >65: Increase	Adjusted	Associated with psychosocial and organizational factors at work
				Pain in arms		Prevalence: Decrease	Prevalence: variable. Decrease until 54 years, 55 – 64: variable >65: Increase		
Großschädl et al, 2014 (28) Austria	Household	1973–2007 (34 years)	General population 64 052 individuals	Back pain	Self-reported	Untreated	Prevalence: increase	Standardization	Linked to workload, sedentary activities, BMI and obesity, evolving perception of pain, and cultural changes
Martin et al, 2014 (49) US	Household	1997–2010 (13 years)	General population ≥40 years 78 328 Individuals	Back pain	Questionnaire	Untreated	Prevalence: variable	Adjusted	Linked with BMI & obesity
			Neck pain		Untreated	Prevalence: variable		
				Other MSD		Untreated	Prevalence: variable		
Jimenez-Sanchez et al, 2010 (50) Spain	Household	1993–2006 (13 years)	General population 92 893 individuals	Invalidating MS pain	Self-reported	Prevalence: variable	Prevalence: Bell curve (peaked in2001)	Stratified	Absence of hypothesis
Leijon et al, 2009 (51) Sweden	Household	1990–2006 (16 years)	General population 63 876 individuals	Low back pain	Self-reported	Untreated	Prevalence: variable	Direct standardization	Linked to increased professional or economic pressure and/or resulting from cultural changes (in media)

**8 time-series studies**									

Ackerman et al, 2019 (52) Australia	Medical records	2003–2013 (10 years)	General population	Hip arthro-plasties	Medical exam	Untreated	Incidence: increase	Stratified ages	Linked with BMI & obesity
				Knee replacements		Untreated	Incidence: increase		
Gelfman et al, 2009 (53) US	Medical record	1981–2005 (24 years)	General population (Olmsted County, Minnesota)	Carpal tunnel syndrome (CTS)	Medical records	Untreated	Incidence: increase	Direct standardization	Greater awareness of CTS among the general population and increasing proportion of at-risk occupations
Holte et al, 2003 (30) Norway	Administrative records (pensions)	1968–1997 (29 years)	General population	Disability pension: RA, OA, soft tissue rheumatism	Medical exam	Untreated	Incidence: increase among women, bell-shaped among men (peaked in the 80s)	Stratified ages	Linked to an increased general demand for fitness or changes in pain perception explained by cultural changes
Paloneva et al, 2015 (33) Finland	Hospital record & surgery	1998–2011 (13 years)	General population	Open and arthroscopic rotator cuff repair	Medical exam & surgery	Untreated	Incidence: increase	Stratified ages	Medical and technical advances leading to improved access to diagnosis and surgery
Pekkala et al, 2017 (26) Finland	Admin- istrative records (sickness insurance)	2005–2014 (9 years)	General population (25-64 yrs.)	Sickness absence due to MSD	Medical exam	Untreated	Prevalence: decrease	Adjusted	Probably linked to the alleviation of the physical demands of the work and better occupational health safety
Spitaels et al, 2020 (27)Belgium	General practitioners (primary aid) from a network of registers.	1992–2013 (21 years)	General population	Knee osteoarthritis	Medical exam	Incidence: U shape Prevalence: increase	Incidence: U shape Prevalence: increase	Standardized and stratified	Linked to BMI & obesity, better access to diagnosis, surgery and preventive medicine, and cultural changes (in patients and practitioners)
Swain et al, 2020 (54) UK	GP hospitals medical record	1997-2017 (20 years)	General population	Osteoarthritis	Medical exam	Incidence: bell curve Prevalence: augmentation	Incidence: bell curve (peaked in 2004-2005) Prevalence: increase	Direct standardization and ACP	Cultural changes in practitioners; cohort effect among people born after the 1960s, who may be less exposed to very physically demanding occupations
Yu et al, 2017 (31) UK	Medical records (primary care)	1992-2013 (21 years)	General population	Clinical osteoarthritis	Medical exam	Variable	Incidence: increase	Standardized and ACP	Similar trends in obesity, a risk factor for OA, and the increased reporting of painful symptoms

a Taking into account the age

The recruitment of individuals for repeated cross-sectional studies was carried out from household-based sampling designs. Among cohort studies, 1 article relied on the recruitment of hospital-based participants, 1 recruited participants from occupational health records, and 1 recruited participants from previous surveys completed at home. Most of the time-series studies relied on hospital-based surveillance systems (5 out of 8 articles).

Of the articles included, 5 defined MSD based on pain, and 11 defined them according to a disability. These 11 articles relying on a disability-related MSD definition were conducted in the Scandinavian countries, the UK, and Australia. Among those, the site of MSD was not specified for 8 articles, 2 articles considered MSD affecting the inferior limbs and the back, and 1 considered MSD affecting the superior limbs.

### Risk of bias and quality of the studies

The studies selected were mainly carried out on the general working population, and the risk of selection bias was considered low or probably low for all studies. Overall, study participants were carefully selected based on a well-defined sampling strategy based on random selection from a national longitudinal or cross-sectional survey.

We considered MSD based on medical diagnosis to be reliable. We classified both MSD based on the medical diagnosis and/or disability at low risk of bias. MSD defined based on pain were classified as a probably low risk of bias. For the studies which administered a questionnaire, we considered that they probably had low bias since it is a good method for detecting chronic pain and disability in the individuals recruited. Studies dealing with temporal trends in MSD by controlling for age and then for other factors were considered at low risk of bias for the confounding factors. Studies not taking other potential confounders were considered likely to be at low risk of bias since here we are only looking at temporal trends in MSD. In the included studies, most of the study authors did not declare a conflict of interest, nor did they receive any support from a company suggesting that there could be a financial interest in the results. Therefore, we assessed these studies as having a low risk of bias in this area. For the studies not clearly mentioning it in the paper, we verified that all the authors were affiliated with public (research) agencies or scientific institutions and, when this was the case, we considered that the studies had a low probability of bias. We did not identify any other biases and therefore assessed all studies as having a probable low risk of other biases.

Of 16 studies, 9 included either tests for temporal trends or confidence intervals (CI) for each value of MSD incidence or prevalence over time. These studies were considered to be of satisfactory quality (trends tests, or Chi-squared), or of probable satisfactory quality (95% CI for MSD incidence and/or prevalence). The studies without statistical tests were considered to be of a probable unsatisfactory quality. In general, we did not identify studies where there was a high risk of bias, or where the quality was too low to justify an exclusion from the review (table 2). Additional results regarding classification of bias and evaluation of statistical methods are provided in supplementary material S4.

**Table 2:** Summary of risk of bias and quality across studies on temporal trends of musculoskeletal disorders (MSD). [L=low; PL=probably low; PH=probably high; H=high; SQ=satisfactory quality; PSQ=probable satisfactory quality; PUS=probable unsatisfactory quality].

	Bias in selection of study participants	Bias due to misclassification of MSD	Bias due to poor consideration of confounding factors	Bias due to conflict of interest	Other biases	Quality of the statistical trend tests
Ackerman et al, 2019 (52)	L	L	L	L	PL	PUS
Dick et al, 2020 (32)	L	PL	L	L	PL	PSQ
Gelfman et al, 2009 (53)	L	L	L	PL	PL	PSQ
Großschädl et al, 2014 (28)	L	PL	L	L	PL	PUS
Guido et al, 2020 (29)	L	PL	L	L	PL	SQ
Holte et al, 2003 (30)	L	L	L	PL	PL	PUS
Jimenez-Sanchez et al, 2010 (50)	L	PL	L	PL	PL	SQ
Leijon et al, 2009 (51)	L	PL	L	L	PL	PSQ
Martin et al, 2014 (49)	L	PL	L	PL	PL	SQ
Paloneva et al, 2015 (33)	L	L	L	L	PL	PUS
Pekkala et al, 2017 (26)	L	L	L	L	PL	SQ
Söderberg et al, 2020 (34)	L	PL	PL	L	PL	PUS
Solomon et al, 2007 (48)	PL	PL	PL	L	PL	PUS
Spitaels et al, 2020 (27)	L	L	L	L	PL	SQ
Swain et al, 2020 (54)	L	L	PL	L	PL	PUS
Yu et al, 2017 (31)	L	L	L	L	PL	PSQ

### Temporal trends of the incidence and prevalence of MSD

Some studies simultaneously reported results for several MSD and/or indicators (prevalence, incidence), such that the 16 included studies included a total of 23 results extracted for this review. Among these results, 9 used a definition of MSD based on pain and 14 looked at impacts on work or social life. Among all results, 12 presented temporal trends in prevalence and 11 in incidence. The variability of the definitions of MSD and body sites studied in the articles of our sample precludes meta-analysis and calculation of pooled estimates of temporal trends. Five studies controlled for age in time trends in MSD by stratification, 4 by adjustment, 3 by standardizations, 3 by direct standardization, and 3 by age-period cohort (see table 1).

**Figure 2:**
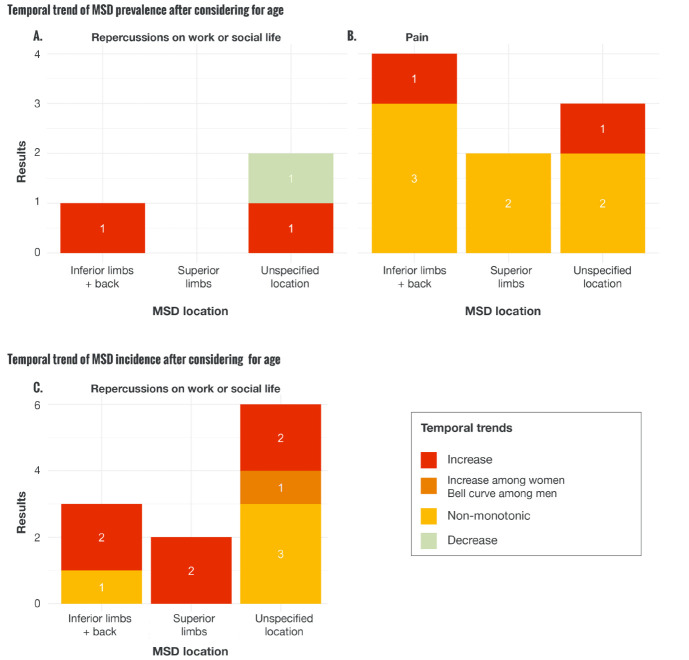
Temporal trends of the incidence and prevalence of MSD according to their location and severity. 2A. Temporal trend of the prevalence of MSD inducing repercussions on work or social life with age considerations. 2B. Temporal trend of the prevalence of pain with age considerations. 2C. Temporal trend of the incidence of MSD inducing repercussions on work or social life with age considerations. Unspecified location refers to MSD that were not associated with a specified body site.

Among the 3 articles defining MSD prevalence based on disability (26), 1 showed that absences due to MSD decreased over time after adjusting for age. Two articles reported an increase in MSD over time, 1 of which reported increases in knee osteoarthritis (27), while the other reported increases in osteoarthritis (28) (figure 2A). Among the 9 results based on pain-related MSD definition, 7 showed non-monotonic change over time, and 2 reported increasing trends [Großschädl et al (28) for lower back pain, and Guido et al (29) for pain in all locations] (figure 2B). These results demonstrate heterogeneity in MSD time trends, including both increases and non-monotonic changes (table 1).

The temporal evolution of the incidence of MSD causing disability also tended to increase or vary according to their site. Among the 6 articles not specifying MSD location, 3 showed variable trends, 2 reported increases over time and 1 (30) reported sex-specific results, with an increase in women and a bell curve for men. In addition, 2 reported an increase over time in MSD located in superior limbs. For the inferior limbs and the back, 2 articles showed an increase in MSD, and 1 reported a variable evolution of MSD (figure 2C and table 1).

### Synthesized evidence

We note that few articles analyzed temporal variations in MSD while controlling for age.

Controlling or not controlling for age may produce divergent pictures of temporal trends in specific MSD. In the article by Yu et al (31), the raw data suggested non-monotonic changes in osteoarthritis incidence over time, whereas increased incidence was observed when standardizing for age. In the article by Dick et al (32), the unadjusted prevalence of back and hand pain decreased over time. After adjusting for age, decreased prevalence was still observed among people <55 years, but variable time trends were observed for those aged 55–64 years, and an increase was observed for those ≥65 years. Thus, these results highlight potentially distinct impacts of age and time in the occurrence of MSD, at least for older age categories.

Fifteen articles suggested that factors other than aging could explain temporal MSD trends (table 1). Regardless of the trends observed, most articles hypothesed a link between cultural changes around the perception of pain (in both caregivers and patients), and a better knowledge of pathologies with improvements in detection and treatment techniques (29–33). We also noticed that five articles related the trends they reported in MSD to similar temporal trends in obesity and body mass index. Additionally, Dick et al (32) suggested that changes in psychosocial and organizational factors at work could explain the non-monotonic trends that they observed between 2002 and 2014. Söderberg et al (34) suggested that the non-monotonic trends they reported reflected changes in disability eligibility criteria rather than in the underlying exposures. Finally, the only article which reported a decrease in the instances of sick leave due to MSD (26) explained it by a probable reduction in the physical demands of work and better health and safety at work.

## Discussion

This literature review identified a limited number of articles reporting temporal trends in MSD while controlling for age. Study duration ranged from 10–55 years, which allowed for longitudinal analysis of MSD occurrence. Temporal trends in MSD varied according to the site of the MSD, the criteria used to define MSD (either associated with pain and/or a disability), and the indicator used (prevalence or incidence). We observed temporal heterogeneity in the occurrence of MSD considered, with mainly non-monotonic or increasing trends reported. Of note, based on studies reporting both crude and age-controlled indicators, we observed that accounting or not accounting for age could lead to diverging temporal trends, at least among the highest age categories.

This literature review identified some important gaps and residual uncertainty in the evidence currently available. First, although our inclusion criteria were broad, the systematic review only identified studies conducted in Western, high-income countries: USA, Europe (especially Scandinavian countries), and Australia. This lack of evidence considering the burden of MSD and their socioeconomic implications does not allow us to provide an interpretation of the evolution of MSD among the global working population (35).

Occurrence of the different groups of MSD considered in this review (pain versus disability) varied over time depending on the indicator considered (prevalence versus incidence). To diagnose the occurrence of MSD, several scales allow for quickly and easily assessing pain intensity (visual analog pain scale, simple numeric scale, simple verbal scale) (36, 37). It is important to note, however, that although the validity of these diagnostic tests is comparable in educated patients, those who are less or uneducated may be led to answer differently. These scales do not allow for a complete assessment of the pain component, but they can allow for repeated self-assessments since they are very quick to complete. It is also possible that the pain reported by patients responds more quickly to changes in working conditions or other factors (such as cultural changes) than do longer disabling pathologies. Therefore, we must remain cautious about our interpretations of temporal changes in the occurrence of MSD, depending on whether the observed outcome relates to self-reported pain or more disabling pathologies diagnosed by doctors (38). Pain classification measurements must therefore include aspects such as the severity, frequency, and intensity of pain as well as measurements of changes in working conditions (39).

We hypothesize that observed heterogeneity in temporal trends of MSD occurrence results from temporal heterogeneity in the evolution of MSD risk factors in different populations. In most of the countries covered by this review, a fundamental change in the tertiarization of work has been observed, resulting in an overall reduction in occupational physical constraints (40, 41). However, a reduction in MSD is not systematically expected from decreased exposure to biomechanical factors. The analyses from the ESTEV survey (42) show in particular that the viscoelastic nature of periarticular soft tissues can also play a role in the occurrence of low back pain. Thus, prolonged exposure from carrying heavy loads can potentially cause an irreversible deformation of these tissues (“memory of the exposure” or “creep phenomenon”), which may explain the fact that, in older age groups, some MSD have not decreased despite decreased biomechanical exposures. Moreover, a decrease in occupational physical constraints may have arisen concomitantly with an increase in work-related mental load, which can also play a significant role in the occurrence of MSD (8, 43).

The main limitation of this review results from the fact that we exclusively searched electronic bibliometric databases of scientific literature. This means that we did not consult the gray literature or governmental reports on MSD that were not peer-reviewed by external readers. Another limitation is that, since we used the generic term musculoskeletal disorders/disease as a keyword, it is possible that we missed articles on specific MSD that did not mention the term MSD in the abstract or key terms. Lastly, variability in MSD definitions and body sites among our study sample prevented us from conducting a meta-analysis and computing pooled estimates of time trends.

Finally, we do not have studies capturing MSD data during the health crisis linked to the COVID-19 pandemic. This sanitary situation could possibly be at the origin of the evolution and emergence of certain professions which can potentially be at the origin of changes in the occurrence of MSD (telework and bad postures, sedentary habits, intensification of work, work on task linked to a digital platform, increased deliveries carrying heavy loads at reduced times, stress, etc.) (44–46). In the future, longitudinal data that can capture this information could be an interesting addition to the interpretation and understanding of the occurrence of MSD over time (47).

### Concluding remarks

To our knowledge, this is the first systematic search and review of studies reporting on MSD occurrence while accounting for the key confounding impact of age. Our findings suggest disparity in the literature regarding the temporal evolution of MSD occurrence, depending on the site of the MSD and whether accounting for MSD defined by scales of self-reported pain or disability. Overall, studies controlling for age reported either non-monotonic changes or increases in MSD occurrence over time. Factors other than aging that have also been suggested to underlie temporal changes in MSD occurrence include changes in obesity, occupational and cultural exposures, and pain tolerance. The current body of evidence, however, highlights residual uncertainties, especially given the limited number of articles on this subject and the fact that we only found articles in wealthy countries. Notably, this review demonstrates the type of research and data that are lacking to anticipate the temporal trends in the MSD occurrence, which is an important question in terms of prevention. We also showed that in the included articles, the temporal trends of MSD varied mainly between increase and non-monotonic changes depending on their site, severity, and age.

## Supplementary material

Supplementary material
